# Milk microbiomes of three great ape species vary among host species and over time

**DOI:** 10.1038/s41598-022-15091-z

**Published:** 2022-06-30

**Authors:** Sally L. Bornbusch, Mia M. Keady, Michael L. Power, Carly R. Muletz-Wolz

**Affiliations:** 1 Center for Conservation Genomics, Smithsonian’s National Zoo and Conservation Biology Institute, Washington, DC USA; 2 Center for Species Survival, Smithsonian’s National Zoo and Conservation Biology Institute, Washington, DC USA; 3 Nelson Institute for Environmental Studies, University of Wisconsin-Madison, Madison, WI USA

**Keywords:** Microbial ecology, Zoology, Bacteria, Microbial communities

## Abstract

In mammalian neonates, milk consumption provides nutrients, growth factors, immune molecules, and microbes. Milk microbiomes are increasingly recognized for their roles in seeding infant gut microbiomes and priming immune development. However, milk microbiome variation within and among individuals remains under investigation. We used 16S rRNA gene sequencing to investigate factors shaping milk microbiomes in three captive great ape species: *Gorilla gorilla gorilla* (individuals, N = 4; samples, n = 29), *Pongo abelii* (N = 2; n = 16), and *Pongo pygmaeus* (N = 1; n = 9). We demonstrate variation among host species, over lactation, and between housing facilities. In phylogenetic community composition, milk microbiomes were distinct among the three ape species. We found only a few shared, abundant bacterial taxa and suggest that they likely serve functional roles. The diversity and community composition of milk microbiomes showed gradual changes over time in gorillas and the Bornean orangutan, which was detectable with our comprehensive sampling over lactation stages (> 300-day span). In gorillas, milk microbiomes differed between housing facilities, but were similar between dams within a facility. These results support the strong influence of evolutionary history in shaping milk microbiomes, but also indicate that more proximate cues from mother, offspring, and the environment affect the distribution of rarer microbial taxa.

## Introduction

All mammals are born obligate lactivores, with milk serving as a formative force for processes ranging from digestion and metabolism to immune and nervous system development^1,2^. Increasingly, milk is also recognized as one of many ways by which microbes are transferred vertically from mother to infant^3,4^. Vertical microbial transmission is an important seeding event that helps shape the infant’s own microbial communities and immune responses^3,5^. The communities of bacteria in milk, collectively known as milk microbiomes, can stem from numerous sources, including the mammary gland, maternal gut, and maternal skin microbiomes^6^. These maternally sourced microbes can be transmitted to the infant through milk consumption^7^. Hypotheses on the functions of these milk microbes include helping the infant digest milk oligosaccharides and other biomolecules^8,9^, training the infant immune system to recognize and possibly tolerate commensal or symbiotic microbes^10,11^, and, overall, providing the foundations for infant gut microbiomes^3,12^. The ubiquity of milk and its associated microbes across the mammalian tree, along with milk’s importance to infant success warrant further research into milk microbiome variation. Here, we investigate cross-sectional and longitudinal variation in milk microbiomes of three relatively closely related nonhuman primates (NHPs): Western lowland gorillas (*Gorilla gorilla gorilla*), Sumatran orangutans (*Pongo abelii*), and a Bornean orangutan (*Pongo pygmaeus*). With the resolution to examine intra- and inter-specific patterns over time, our study expands frameworks for understanding how milk microbiomes are shaped in NHPs. Beyond adding to our understanding of how milk microbiomes are structured across mammalian species, the results of this study have the potential to inform the reproductive care and husbandry of captive great ape populations; characterizing baseline microbial communities associated with successful lactation and infant development can inform diagnostic tools for reproductive dysfunction and can be applied to the formulation microbial therapies such as pre- and probiotics.

Lactation strategies vary among mammalian taxa, with the duration of lactation spanning from 4 days in hooded seals (*Cystophora cristata*)^13^ to upwards of eight years in orangutans (*Pongo abelii* and *Pongo pygmaeus*, two of the species included in this study^14^). These strategies are reflected in the macronutrient content of milk—namely protein, sugar, and fat—and the associated gross energy, which vary across species and across lactation phases^15,16^. Primates, in general, produce milks that are relatively low in fat and protein and higher in carbohydrates and water content, reflecting relatively long lactation periods, slow-growing infants, and short inter-nursing intervals^17–19^. In zoo gorillas and orangutans, the composition of milk nutrients and bioactive molecules is most variable during early and late lactation, but is relatively stable during mid lactation (i.e., 2–18 months postpartum in gorillas^20^ and 6–18 months in orangutans^21^). We have previously shown that nutritional composition correlates with components of NHP milk microbiomes^22^, highlighting potential relationships between milk nutrients and bacteria.

Most widely reported in humans and domestic animals, the microbes in milk originate from multiple maternal body sites^23,24^, can show shifts in composition over time^22,25–27^ and vary among mammalian taxa^22,28^. Maternal gut bacteria appear able to travel along the entero-mammary pathway, enter the mammary gland, and subsequently be transferred to the infant via milk^29,30^. Additionally, there can be bidirectional transfer of microbes between the mammary gland and infant oral cavity^24,31^. In humans, the dominant bacteria of milk microbiomes include members of the Firmicutes phylum, namely, *Staphylococcus*, *Streptococcus*, and *Lactobacillus* ^32,33^. The microbial taxa that comprise the human milk microbiome shift over time, from *Staphylococcus* and *Streptococcus* spp. dominating the communities in prebirth colostrum and early lactation milk to more diverse communities in later lactation^32,34^. Temporal patterns of diversity in human milk microbiomes vary across individuals with some showing colostrum having greater bacterial diversity compared to later samples whereas others remain stable over time^35,36^. In other animals, milk microbiomes primarily differ from colostrum or early lactation compared to mid and late lactation, potentially reflecting different stages of maternal investment and infant development^25,37^. However, previous studies have been focused on variation between discrete lactation phases; greater longitudinal resolution that does not rely on binned lactation stages is needed to identify more nuanced variation in milk microbiomes over time.

Next steps towards a more comprehensive understanding of milk microbiomes and their roles in host health and development should include investigations of variation across mammalian taxa. Nonhuman primates (NHPs), particularly great apes, are excellent models for investigating milk microbiomes given their ability to voluntarily provide milk samples, close evolutionary relationship with humans that enable applied value to evolutionary medicine and human health (e.g., formula and probiotic synthesis), and conservation concern with many species nearing extinction^38^. We suggest that understanding milk microbiomes may be similarly important for improving reproductive success in ex-situ NHP populations.

Although longitudinal analyses of NHP milk microbiomes are sparse, previous studies have demonstrated changes in NHP milk over time. In contrast to the patterns seen in humans, in vervets monkeys (*Chlorocebus aethiops sabaeus*), milk microbiome diversity was greater in later lactation compared to early lactation^37^. In a longitudinal study of gorilla milk microbiomes performed by our group, milk microbiome bacterial richness changed over time, but with no consistent pattern, while bacterial composition generally became more dissimilar over time^22^. The present study builds on the results of our previous study by further investigating longitudinal patterns in NHP milk microbiomes with a greater sampling of all individuals over lactation time and comparing differences in longitudinal patterns among host species.

To investigate variation within and among host species and over time, we used 16S rRNA gene sequencing to determine the diversity, composition, and membership of the milk microbiomes of gorillas and orangutans housed under human care at different facilities. The factors that drive milk microbiomes are likely numerous and complex, and include variables that are both endogenous (e.g., species identity and physiology) and exogenous (e.g., environmental setting and diet) to the host. In this study, our objectives were to assess the relative influences of host species and time (i.e., day postpartum), and make preliminary assessments of the influence of housing facility in gorillas, the only species for which we have individuals housed at different facilities. Broadly, we expected that host species would be the strongest driver of milk microbiomes and would underlie foundational differences in microbiome composition and membership^22^. We expected the more closely related two orangutan species to have more similar milk microbiomes compared to gorillas. Based on the results of our group’s previous studies of ape milk nutrient composition and microbiomes^22^, we expected ape milk microbiome diversity to neither decrease nor increase over time. However, we predicted that the composition of an individual’s milk microbiome would change over time, indicating shifting microbial membership. Among members of the same host species, we expected the compositional shifts to be similar in magnitude and reflect temporal changes in the same bacterial taxa. In regard to housing facility, we predicted that members of the same host species (i.e., gorillas) housed at different facilities would be similar in the abundant microbial taxa, but would differ in less abundant microbes that reflect environmental or dietary variation^35,39,40^.

## Methods

### Subjects and sampling protocols

**Figure 1:**
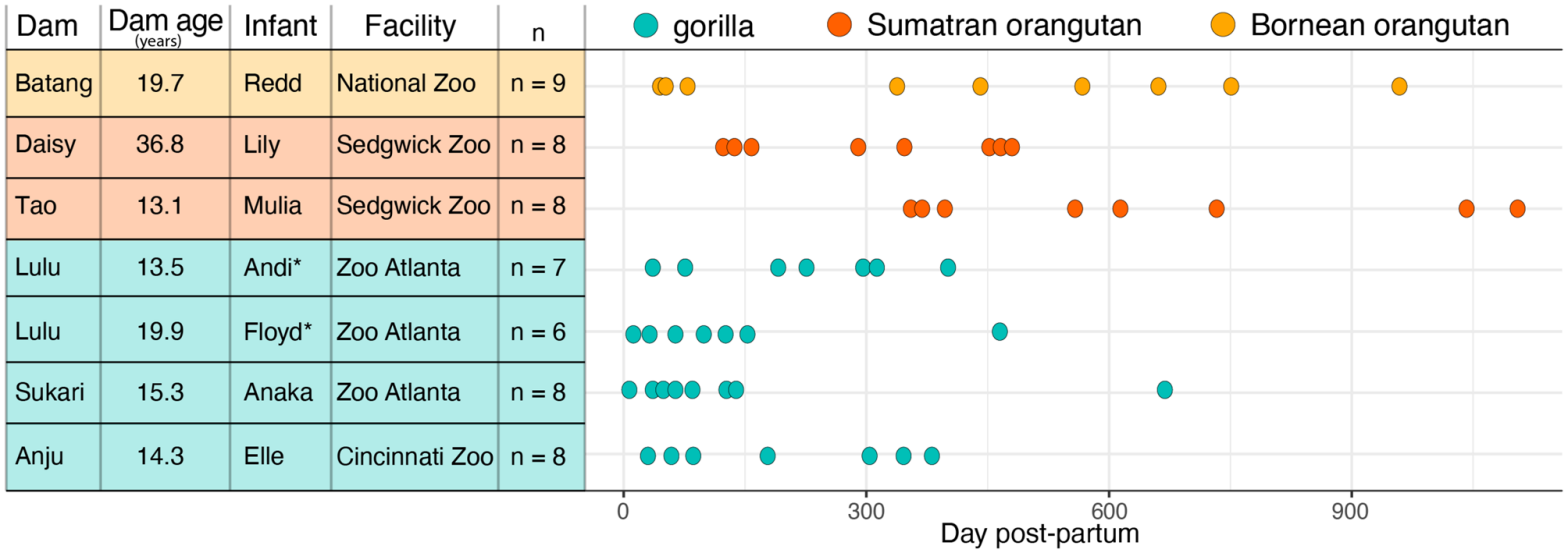
Schematic of sample metadata and longitudinal sampling, colored by species. Includes Dam ID and age (in years), Infant ID, housing facility, the number of milk samples (n) collected within a dam’s lactation, and the longitudinal resolution of sample collections (days postpartum). Infants with an asterisk are subsequent infants from the same dam.

Study subjects were six female great apes from three species, sampled across the nursing of seven infants (Fig. 1): Western lowland gorilla (*Gorilla gorilla gorilla*: n = 3 dams, 4 infants), Sumatran orangutan (*Pongo abelii*; n = 2 dams, 2 infants), and Bornean orangutan (*Pongo pygmaeus*; n = 1 dam, 1 infant). Only dams free of mastitis were included in the study. Individuals were housed at one of four facilities in the U.S. (Fig. 1): Cincinnati Zoo (one gorilla), Zoo Atlanta (two gorillas), Sedgewick County Zoo (two Sumatran orangutans), and Smithsonian National Zoological Park (one Bornean orangutan). Provided diets were similar across individuals and facilities, complying with nutritional standards for each species. Zoo gorillas and orangutans generally have similar lactation strategies and nursing duration in captivity^20,21^. While there is intra-individual and inter-species variation, early lactation stage can generally be considered from 0–3 months, mature milk lactation from 3 to 18+ months, and late lactation generally the month prior to weaning, which, at the extremes, can be up to 8 years in some wild orangutans^14^. The majority of our samples were within the early (~ 0–3 months) or mature (~ 3–18 months) lactation stages, except for two orangutans that had some samples in what is likely the late lactation stage (900+ days). We were limited in what milk samples we had from the same mothers over lactation, but we designed a strategy to have at least six time points for each female that either captured early and mature lactation stages or captured multiple time points over a 300+ day range (Fig. 1). Because we did not have nutritional or behavioral data in this study, which are generally used to determine lactation phase^20,21^, we used ‘day postpartum’ as our longitudinal variable because of its independence from other factors. In addition, to minimize the issue of different time periods sampled, we chose to examine longitudinal trajectories by fitting measures of microbial diversity and composition over time as linear relationships as opposed to discrete, binned lactation stages (see “Bioinformatics and statistics” below).

All subjects were trained to provide milk samples, allowing for voluntary sample collection via manual expression of the mammary gland by facility staff. Samples were collected during routine training and enrichment sessions that were scheduled and performed by facility staff. Staff wore appropriate personal protection equipment, including sterile glove, for all collections. The nipple area was not sterilized prior to collection; we acknowledge the potential for environmental sources of microbes, but those should have minor contributions compared to microbes in the targeted milk sample. Samples were collected between day 7 and 1105 postpartum, with varying duration and frequency across individuals (Fig. 1). Total collected sample volume ranged from 1 to 25 ml. Samples were homogenized prior to aliquoting for microbiome analyses so that each aliquot was a representative mixture of all milk collected. Milk samples were collected in sterile tubes, immediately placed on ice, and stored frozen until shipment on dry ice to the Smithsonian Nutrition Lab for analysis. Upon arrival, samples were aliquoted into sterile cryovials and stored in a − 80 °C freezer until extraction.

Experimental design and sampling protocols followed applicable Animal Research: Reporting of In Vivo Experiments (ARRIVE) guidelines and the Principles for Ethical Treatments of Nonhuman Primates set by the American Society of Primatologists. The project was approved by the Ape Taxon Advisory Group of the American Association of Zoos and Aquaria and was also approved by the Research Committees of the Cincinnati Zoo, Zoo Atlanta, Sedgwick County Zoo, and Smithsonian National Zoological Park. There were no in vivo animal experiments in this project.

### DNA extraction, preparation, and sequencing

DNA was extracted from all samples (n = 55) using DNeasy PowerSoil Pro kit (Qiagen, Germany). We followed the manufacturer’s protocol with minor modifications to improve DNA yield from typically low-yield sample types. We used 500µL of milk and centrifuged samples at 10,000×*g* for ten minutes at room temperature. The supernatant was discarded and the remaining pellet was used for extraction. To improve DNA yield, we included additional incubation steps prior to bead-beating to dissolve the pellet (65 °C for 10 min at 40 rpm) and prior to the final elution (Solution C6 warmed to 60 °C and added to samples for a 5-min incubation).

We prepared 16S rRNA gene metabarcoding libraries for biological samples, positive controls (ZymoBIOMICS microbial community standards; Zymo Catalog No. D6300 & D6305) and negative controls (extraction and PCR negative controls) using a two-step PCR library preparation procedure with primers for the V3–V5 region (515F-Y and 939R) following previously reported methods^41^. We pooled the cleaned libraries in equimolar ratios and sequenced them on one Illumina MiSeq run (2 × 300 bp) at the Center for Conservation Genomics (Washington, DC).

### Bioinformatics and statistics

Bioinformatics were performed namely in R Studio^42^ (R ver 4.1.0), with minor processes in QIIME2^43^ (ver 2021.8). Briefly, we used the dada2 package^44^ in R to quality filter and trim sequences, merge forward and reverse reads, remove chimeric sequences, assign taxonomy (DADA2-formatted RDP trainset 18 database)^45^ and generate amplicon sequence variant (ASV) feature tables. We used decontam to remove potential contaminants using the combined method^46^, and removed 33 ASVs identified as potential contaminants. We further filtered sequences identified as non-bacterial (e.g., chloroplasts and mitochondria) and those that had raw counts of less than ten across all samples. In addition to taxonomic identifications provided by the RDP database, we applied NCBI BLAST to query sequences from certain ASVs against NCBI GenBank database to estimate species-level identifications. We used QIIME2 to generate phylogenetic trees which, in combination with ASV tables and metadata, were imported into R for downstream analyses using phyloseq^47^. The ZymoBIOMICS microbial community standards (positive controls) were analyzed and we found genera in similar relative abundances as described by Zymo. Of the 55 samples sequenced for this study, one sample was removed from downstream analyses due to low sequence coverage. After initial sequence data processing, the retained 54 samples yielded 1,609,532 reads, with a mean sequencing depth of 29,803 (± standard deviation = 10,626) reads per sample.

We normalized raw sequence counts and calculated alpha diversity and beta diversity measures. To normalize sequence counts for alpha and beta diversity analyses, we scaled with ranked subsampling in R (SRS)^48^ using a normalization sequencing depth of 6107 reads (Fig. S1). SRS uses a two-step normalization approach that, compared to rarefying, has greater reproducibility and better preserves bacterial diversity and ASV frequencies^48^. We used the normalized counts to calculate three measures of diversity (alpha diversity: Shannon, observed features or ASV richness, and Faith’s phylogenetic diversity) and two measures of composition (beta diversity: unweighted UniFrac (UUF), also referred to as presence-absence composition) and weighted UniFrac (WUF), also referred to as abundance-weighted composition). For visualization purposes only, we used raw sequence counts to calculate the relative abundance of all taxa and included the conglomerate “Other” to represent the rare taxa that had relative abundances < 1%.

We used log-ratio transformations of raw sequence counts to examine abundances of bacterial taxa. To account for the compositional nature of microbiome data, for all statistical analyses of bacterial abundance, we used center log-ratios (CLR) transformations on the relative abundances of all taxa, which reflect log-transformed ratios of the raw sequence counts of each taxon over the geometric mean of all other taxa in the sample^49^.

To characterize variation in bacterial diversity and CLR abundances, we used linear mixed-effects models (LMMs; R, lmer in {vegan})^50^ that included, host species, number of days postpartum (referred to as “day” throughout, and their interaction as fixed effects, and infant as a random effect. Parity and infant sex were initially included in statistical models but were found to be non-significant in all cases and so were removed from the final, parsimonious model. By using sampling day as our LMM longitudinal variable, we were able to analyze and compare linear trajectories of microbial diversity and composition within and between individuals. We used Principal Coordinate Analyses to visualize clustering of both measures of bacterial composition. To test for variation in bacterial composition, we used a PERMANOVA with input of beta diversity distance matrices and with the same variables as described above (R, adonis2 in {vegan}; using blocks to account for infant as a random effect). To further assess changes in bacterial composition over time, we calculated distances (UUF and WUF) between the microbial compositions of the first milk sample of each dam’s lactation period (used as a proxy for a baseline community) and every subsequent sample, allowing us to use the LMM model described above to assess longitudinal distance from baseline. To identify taxa that were differentially abundant between the three species, we used Analysis of Compositions of Microbiomes (R, ancom in {ANCOM}ver. 2.1), which calculates a W-statistic reflecting the number of times the log-ratio of a specific taxon to every other taxon was significantly different between the three host species. We used Kruskal–Wallis tests and Pairwise Wilcoxon rank-sum tests with Benjamini–Hochberg adjustments^51^ to test for variation in alpha and beta diversity measures between specific groups of samples, including species and individuals at different facilities. For analyses of composition among individuals within and between housing facilities, we report the mean (x̄) of pairwise UniFrac distances for each variable and the p-value generated by the Pairwise Wilcoxon rank-sum tests described above. For all analyses, a p-value of less than 0.05 was considered statistically significant. Bioinformatic and statistical pipelines are available in Open Science Framework (Project ID and URL TBD).

## Results

### Microbial membership of great ape milk microbiomes

**Figure 2:**
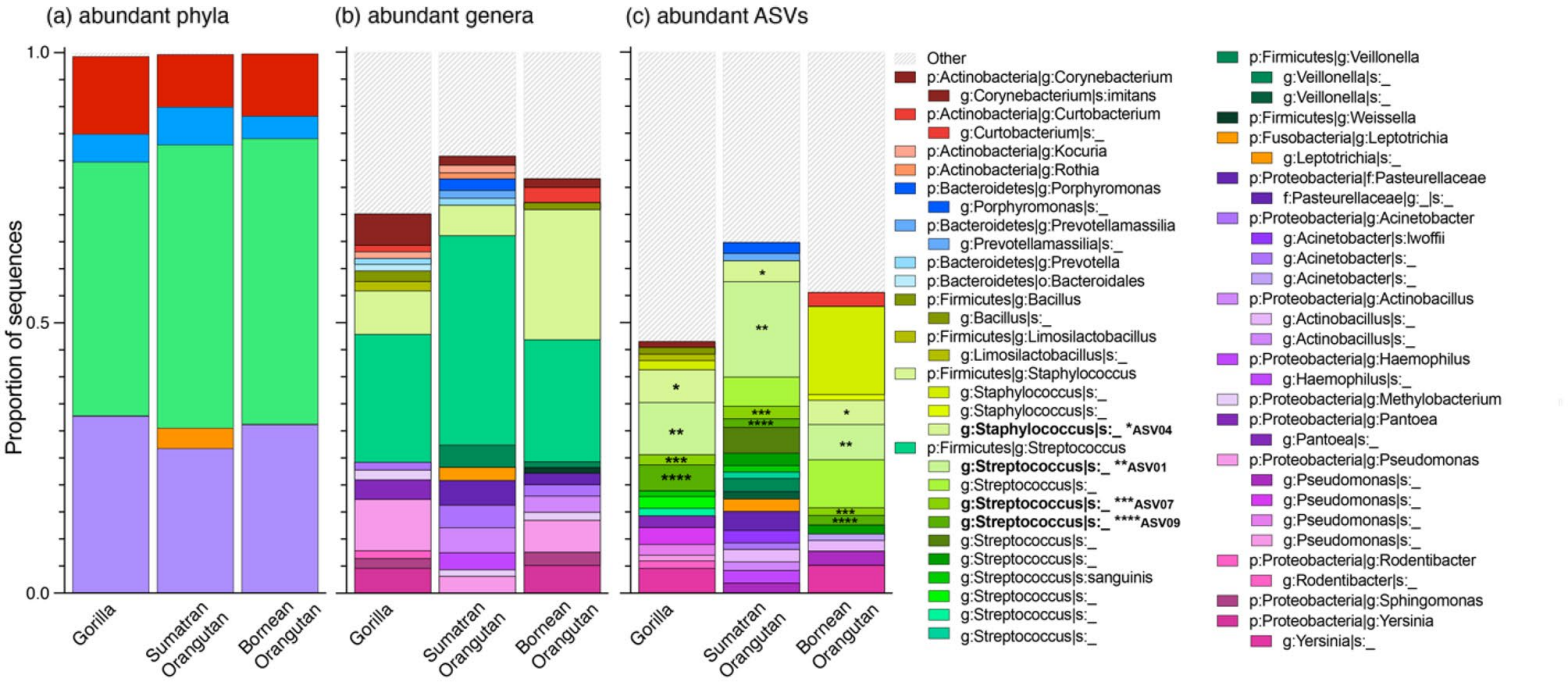
Relative abundances of abundant, bacterial (**a**) phyla, (**b**) genera, and (**c**) ASVs (i.e., accounted for > 1% of the sequences for each species) in the milk microbiomes of gorillas, Sumatran orangutans, and a Bornean orangutan. Taxa are identified by color and, at each of the three taxonomic levels, taxa representing < 1% of the microbiomes were combined into the category “Other”. The four ASVs that are abundant in all three host species are noted with asterisks.

The membership of milk microbiomes of the three ape species included 2,322 ASVs comprising 492 genera in 18 phyla. Across the three ape species, the bacterial membership of milk microbiomes was dominated by members of the Firmicutes (mean relative abundance = 50.72% ± standard deviation = 18.84%), and Proteobacteria (30.24% ± 19.19%) phyla, with smaller contributions from the Actinobacteria (11.88% ± 7.32%), Bacteroidetes (5.43% ± 3.67%), and Fusobacteria (1.33% ± 2.11%) phyla (Fig. 2). At the ASV level, 384 ASVs (16.5% of ASVs) were present in the milk microbiomes of all three host species, which, when combined, accounted for an average of 79.62% ± 12.92% of sequences (64.63% ± 11.67% in gorillas, 69.00% ± 11.25% in Sumatran orangutans, and 97.11% ± 14.47% in the Bornean orangutan), indicating that a small portion of the microbial diversity accounted for a majority of microbial abundance in all samples. In regard to bacterial taxa that differed between host species, ANCOM analysis identified six ASVs that were differentially abundant between the three ape species (Fig. S2).

We found a total of 36 ASVs that were abundant (defined as > 1% of the sequences) in at least one host species. When, combined, the 36 ASVs comprised an average of 59.46% ± 17.15% percent of the milk microbiomes (49.41% ± 16.25% in gorillas, 66.86% ± 11.14% in Sumatran orangutans, and 62.11% ± 18.96% in the Bornean orangutans). There were four ASVs that were abundant in the milks of all three ape species (Fig. 2, noted with asterisks): three members of the *Streptococcus* genus (BLAST majority identifications: ASV01, *S. thermophilus*; ASV07*, S. mitus*; and ASV09, *S. parasanguinis*) and one member of the *Staphylococcus* genus (BLAST majority identification: ASV04, *S. aureus*). These four accounted for, on average, 20.67% ± 16.07% of the milk microbiomes (22.41% ± 18.79% in gorillas, 25.39% ± 12.60% in Sumatran orangutans, and 14.21% ± 9.17% in Bornean orangutans). Two of these four ASVs—*Streptococcus thermophilus* and *Streptococcus mitus*– were present in every sample whereas the *Streptococcus parasanguinis* and *Staphylococcus aureus* ASVs were present in all but one and six of the 54 samples, respectively.

### Variation in milk microbiome diversity and composition across host species

**Figure 3:**
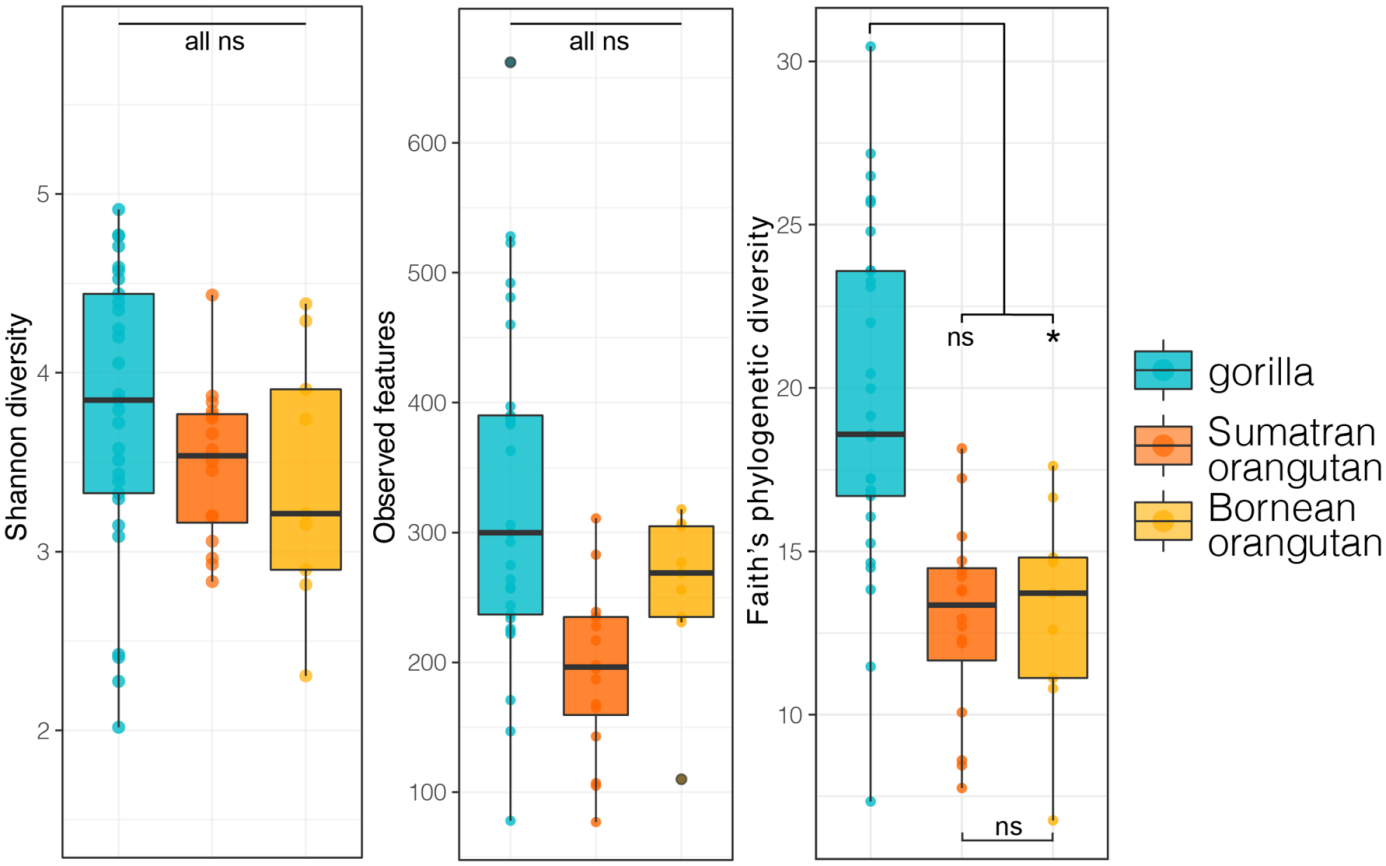
Boxplots of the three metrics of alpha diversity (Shannon diversity, observed (ASV) features and Faith’s phylogenetic diversity), averaged across all samples for gorillas, Sumatran orangutans, or Bornean orangutans. Statistical results calculated with Linear Mixed Models reported in the “Results” (p > 0.5 = ns; p < 0.05*).

**Figure 4:**
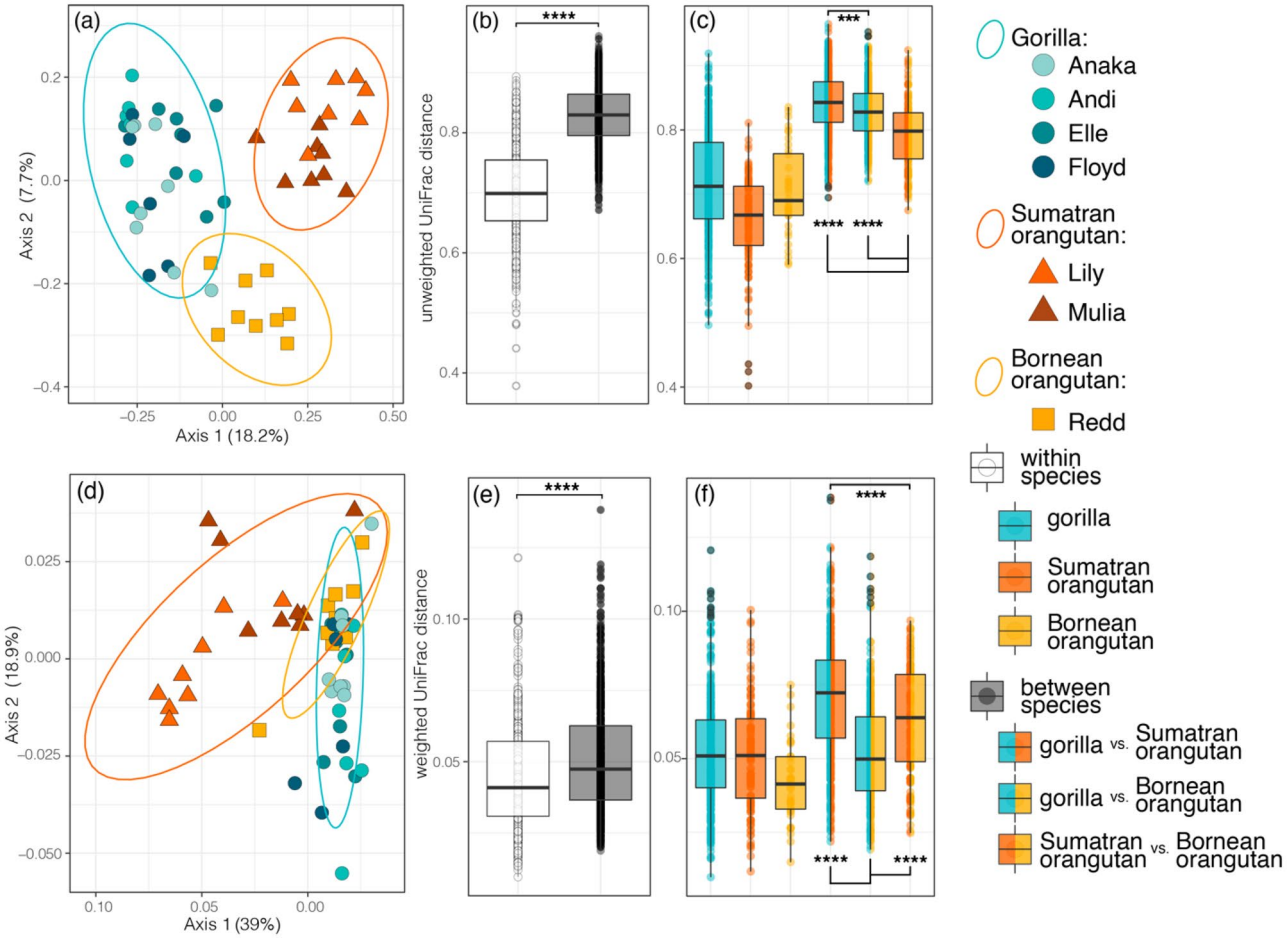
(**a**–**c**) Unweighted UniFrac and (**d**–**f**) weighted UniFrac distances for milk microbiomes received by gorilla, Sumatran orangutan, or Bornean orangutan infants. (**a**,**d**) Principal coordinate analysis with points colored by infant shows clustering by species in coordinate space with 95% confidence interval ellipses. (**b**,**c**,**e**,**f**) Comparisons of pairwise distances within vs. between species with statistical results of Pairwise Wilcoxon Rank Sum Tests with Benjamini–Hochberg adjustment (p < 0.0001****). Tukey-style box and whiskers show the median (center horizontal line) and the interquartile range (upper and lower bounds of the box), with outliers that are 1.5 times less than the 25th quartile or 1.5 times more than the 75th quartile. Statistical results of insterest are shown in the figure; see Table S1 for full statistical results.

Host species was a significant predictor of Faith’s phylogenetic diversity, but not of the other two alpha diversity measures (Fig. 3; LMM: Faith’s phylogenetic diversity, F_2,11.9_ = 4.253, p = 0.040; Shannon diversity, F_2,9.2_ = 1.467, p = 0.279; observed features, F_2,10.5_ = 1.027, p = 0.315). Gorillas had greater phylogenetic diversity than did the Bornean orangutan, but similar phylogenetic diversity to Sumatran orangutans (LMM: gorilla vs. Bornean orangutan, t = 2.825, p = 0.015; gorilla vs. Sumatran orangutans, t = 1.311, p = 0.213). The two orangutan species had similarly diverse milk microbiomes in all three alpha diversity metrics (LMM: Faith’s phylogenetic diversity, t = − 1.360, p = 0.085; Shannon diversity, t = − 1.577, p = 0.147; observed features, t = − 0.431, p = 0.674). Host species was a significant predictor of milk microbiomes composition for both measures of beta diversity (PERMANOVA: UUF, F_2,51_ = 2.661, p = 0.005; WUF, F_2,51_ = 1.821, p = 0.005; Fig. 4). Presence-absence composition (UUF) showed distinct clustering according to host species, whereas abundance-weighted composition (WUF) showed more overlap (Fig. 4a,d), suggesting that variation between species is, in part, driven by less abundant taxa. Individuals within the same species, regardless of facility, were more similar to one another than to individuals from different species (Fig. 4b,e; Kruskal–Wallis test: pairwise UUF distance, χ^2^ = 43.62, p < 0.0001; pairwise WUF distance, χ^2^ = 670.91, p < 0.0001). Gorilla and Sumatran orangutans had distinct milk microbiome composition in both the presence-absence and abundance-weighted measures (Fig. 4c,f; gorilla vs. Sumatran orangutans pairwise distances: UUF, distances μ = 0.841; WUF, μ = 0.070; statistics reported in Table S1). For the other host species comparisons, contrasting patterns were observed between the two measures of bacterial composition. In presence-absence composition, the two orangutan species did not differ in milk microbiome composition (Fig. 4c; Sumatran orangutans vs Bornean orangutan: UUF distances, μ = 0.792; Table S1). In abundance-weighted composition, gorillas and the Bornean orangutan did not differ in milk microbiome composition (Fig. 4c; gorilla vs Bornean orangutan: WUF distances, μ = 0.052; Table S1).

### Longitudinal variation in milk microbiomes

**Figure 5:**
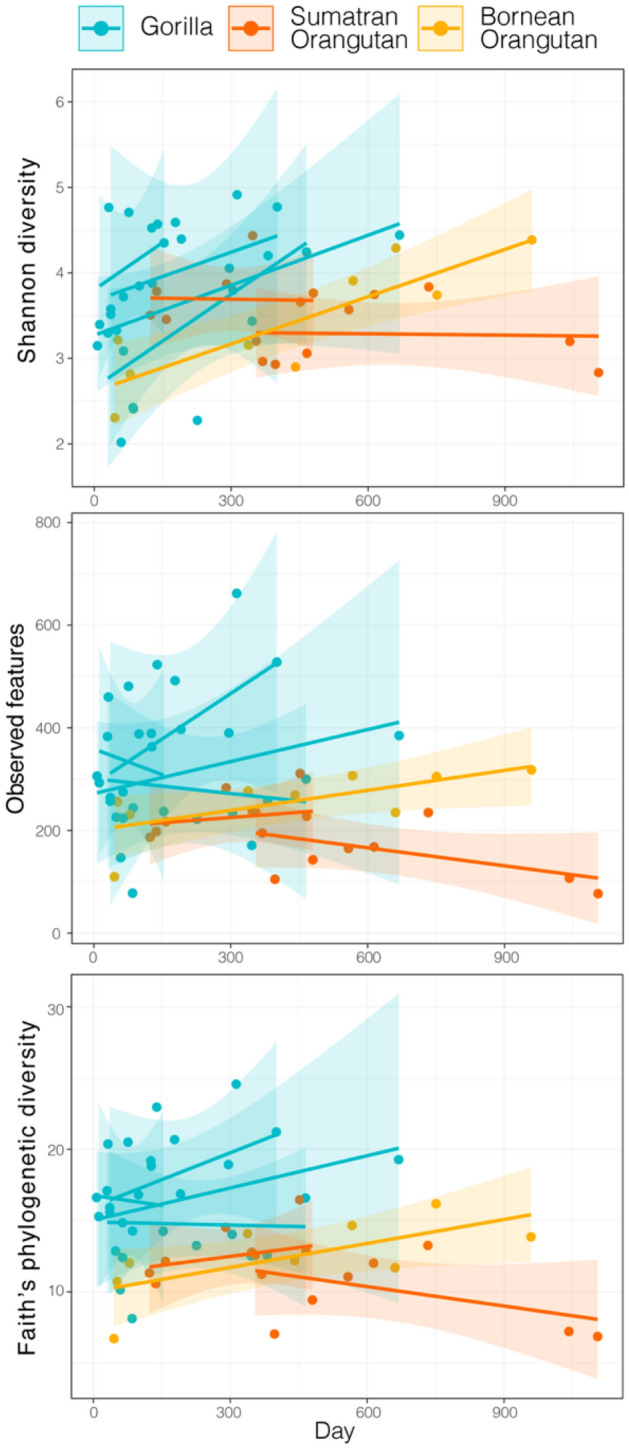
Three metrics of alpha diversity (Shannon diversity, observed (ASV) features and Faith’s phylogenetic diversity) plotted over time for the microbiomes present in the milk of gorillas, Sumatran orangutans, or Bornean orangutan. Lines represent linear regressions for each infant’s longitudinal trajectory with shaded ribbons of 95% confidence intervals. There was a significant day by host species interaction for Shannon diversity (LMM: Shannon diversity, F_2,47.8_ = 3.746, p = 0.030) and day alone was a significant predictor of Shannon diversity, with an increase over time for gorillas and the Bornean Orangutan (LMM: Shannon diversity, F_1,47.9_ = 10.162, p = 0.002). Full results reported in the text.

There were linear longitudinal changes in Shannon diversity, but this depended on host species (day by host species interaction) and was not observed for the other two alpha diversity measures (Fig. 5; LMM: Shannon diversity, F_2,47.8_ = 3.746, p = 0.030; observed features, F_2,47.8_ = 1.816, p = 0.173; Faith’s phylogenetic diversity, F_2,47.0_ = 2.374, p = 0.104). Day alone was also a significant predictor of Shannon diversity, with an increase in Shannon diversity over time, but there was no significant change in the other two alpha diversity measures (Fig. 5; LMM: Shannon diversity, F_1,47.9_ = 10.162, p = 0.002; observed features, F_1,47.9_ = 1.027, p = 0.381; Faith’s phylogenetic diversity, F_1,47.4_ = 2.218, p = 0.142). The day by host species interaction was likely driven by the patterns seen in Sumatran orangutans, which showed little variation over time compared to the other two species (Fig. 5). When the Sumatran orangutans were removed from the model, day became a significant predictor of increases in both Shannon diversity and Faith’s phylogenetic diversity for gorillas and the Bornean orangutan (LMM: Shannon diversity, F_1,32.8_ = 12.630, p = 0.001; observed features, F_1,33.0_ = 1.027, p = 0.381; Faith’s phylogenetic diversity, F_1,33.3_ = 4.363, p = 0.044).

Over time, milk microbiome composition varied with abundant and rare taxa showing different longitudinal patterns. When analyzing change over time between the three species, the interactions between day and host species was significant for both measures of bacterial composition (PERMANOVA: UUF, F_2,51_ = 2.540, p = 0.005; WUF, F_2,51_ = 3.255, p = 0.005), suggesting host-species specific patterns of compositional change over time. Day alone was a significant predictor of presence-absence composition, but not of abundance-weighted composition (PERMANOVA: UUF, F_2,51_ = 1.968, p = 0.045; WUF, F_2,51_ = 1.245, p = 0.080).

**Figure 6:**
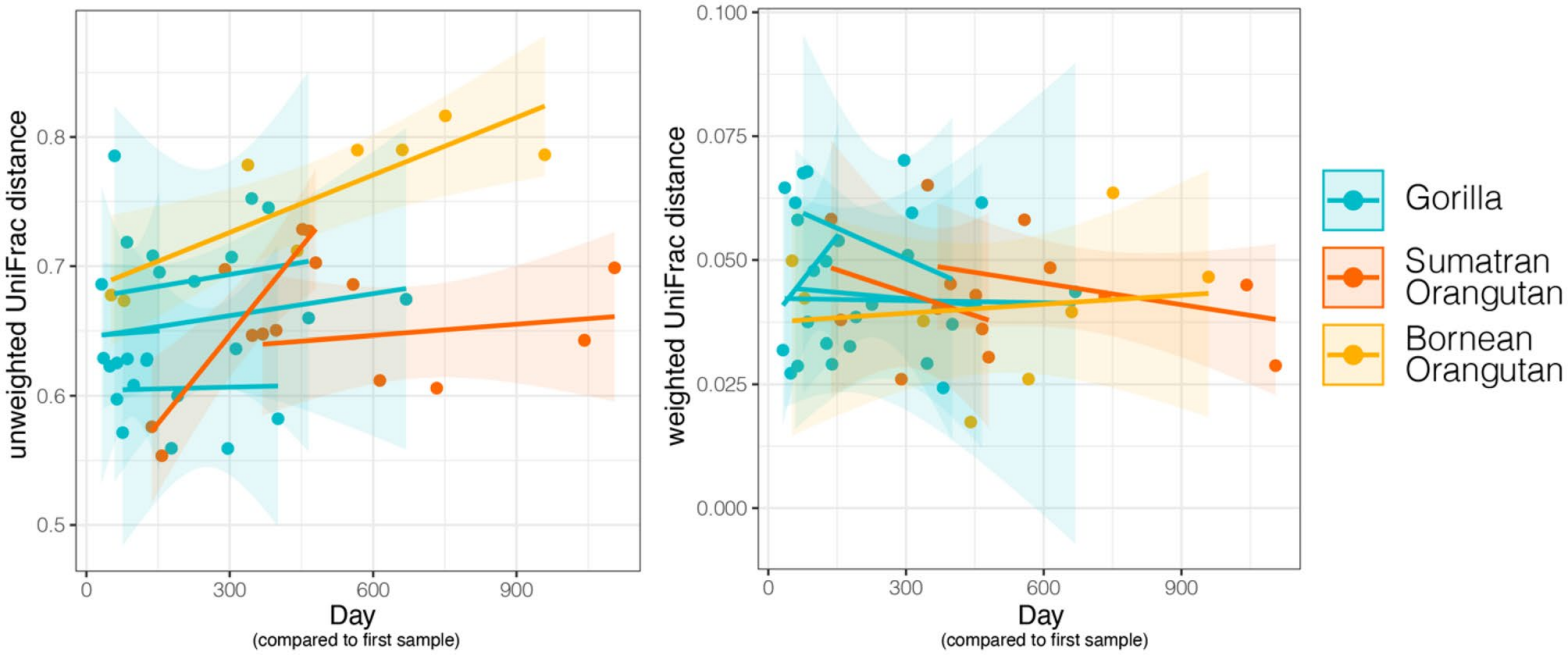
Trajectories of unweighted and weighted UniFrac distances over time for the milk microbiomes of gorillas, Sumatran orangutans, and a Bornean orangutan. Each point represents a distance between each dam's first sample within a lactation and the sample on the respective day. Lines represent linear regressions for the longitudinal trajectory or each lactation with shaded ribbons of 95% confidence intervals. Compared to each individual’s baseline, milk microbiomes became more dissimilar over time for presence-absence composition, but not weighted composition (Linear Mixed Models: unweighted UniFrac, F_1,40.9_ = 6.342, p = 0.015; weighted UniFrac, F_2,41_ = 0.065, p = 0.798).

When analyzing the longitudinal trajectory of each dam's first sample within a lactation as a baseline, milk microbiomes became more dissimilar from baseline over time for presence-absence composition, but not abundance-weighted composition (Fig. 6; LMMs: UUF, F_1,40.9_ = 6.342, p = 0.015; WUF, F_2,41_ = 0.065, p = 0.798), indicating that the abundant taxa remained largely stable while composition and phylogenetic relationships of the less abundant microbes changed over time. All other variables in these linear models of individual change over time (host species and interaction between species and day) were nonsignificant for both beta diversity measures.

**Figure 7:**
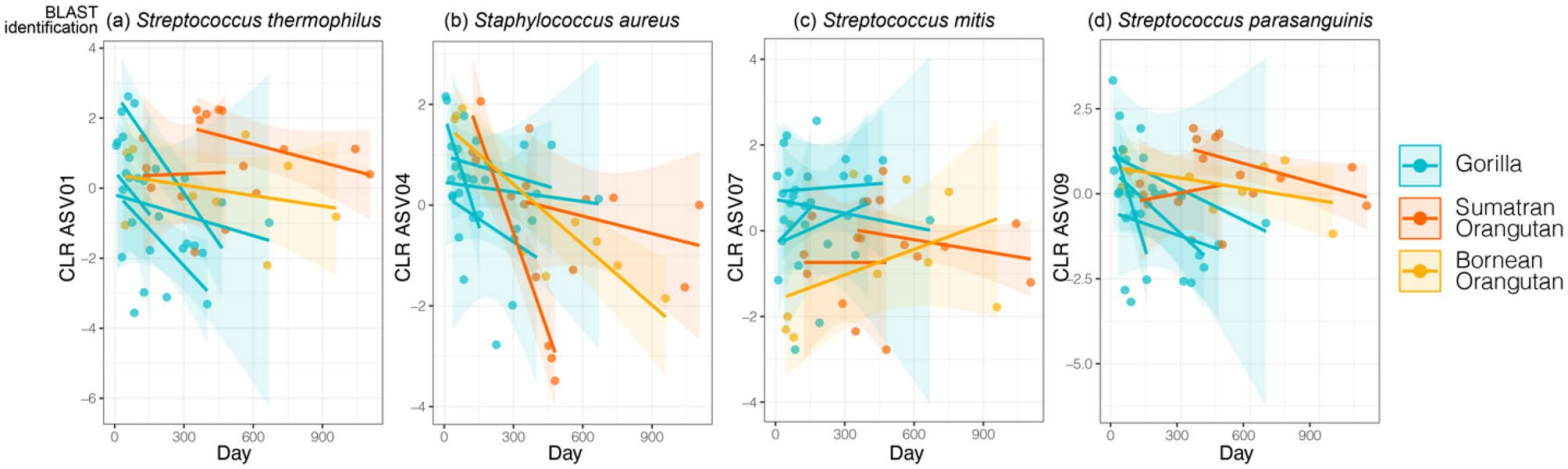
Longitudinal trajectories of centered log-ratios (CLR) of ASV abundance for the four abundant ASVs shared across the milk microbiomes of gorillas, Sumatran orangutans, and a Bornean orangutan. Blast ID is the predicted taxonomic identification, with genus level determined by RDP database and, where possible, species level determined by the majority hit in NCBI GenBank (see bioinformatic methods). Lines represent linear regressions for each infant’s longitudinal trajectory with shaded ribbons of 95% confidence intervals. Statistical results for linear mixed models of ASV abundance are reported in Table S2.

We examined how the 36 abundant bacterial ASVs varied in abundance over time. We found that five bacterial ASVs decreased significantly in CLR abundances over time and two increased significantly over time (Table S2, Fig. S3). For the bacteria that decreased over time, three were shared abundant ASVs, *Streptococcus thermophilus*, *Streptococcus parasanguinis*, and *Staphylococcus aureus* (Table S2; Fig. 7). The fourth shared abundant taxon—*Streptococcus mitus*—remained stable (Table S2; Fig. 7). The day by host species interaction was nonsignificant for three of the four shared ASVs (ASVs 01, 04 and 07; Table S2), indicating that those taxa were responding to time similarly among host species. For ASV09, *Streptococcus parasanguinis*, day by species interaction was significant (Table S2). We found that an additional seven ASVs had a significant day by host species interaction, indicating that these ASVs showed longitudinal changes in at least one of the three host species (Table S2; Fig. S3).

### Variation across housing facilities

**Figure 8:**
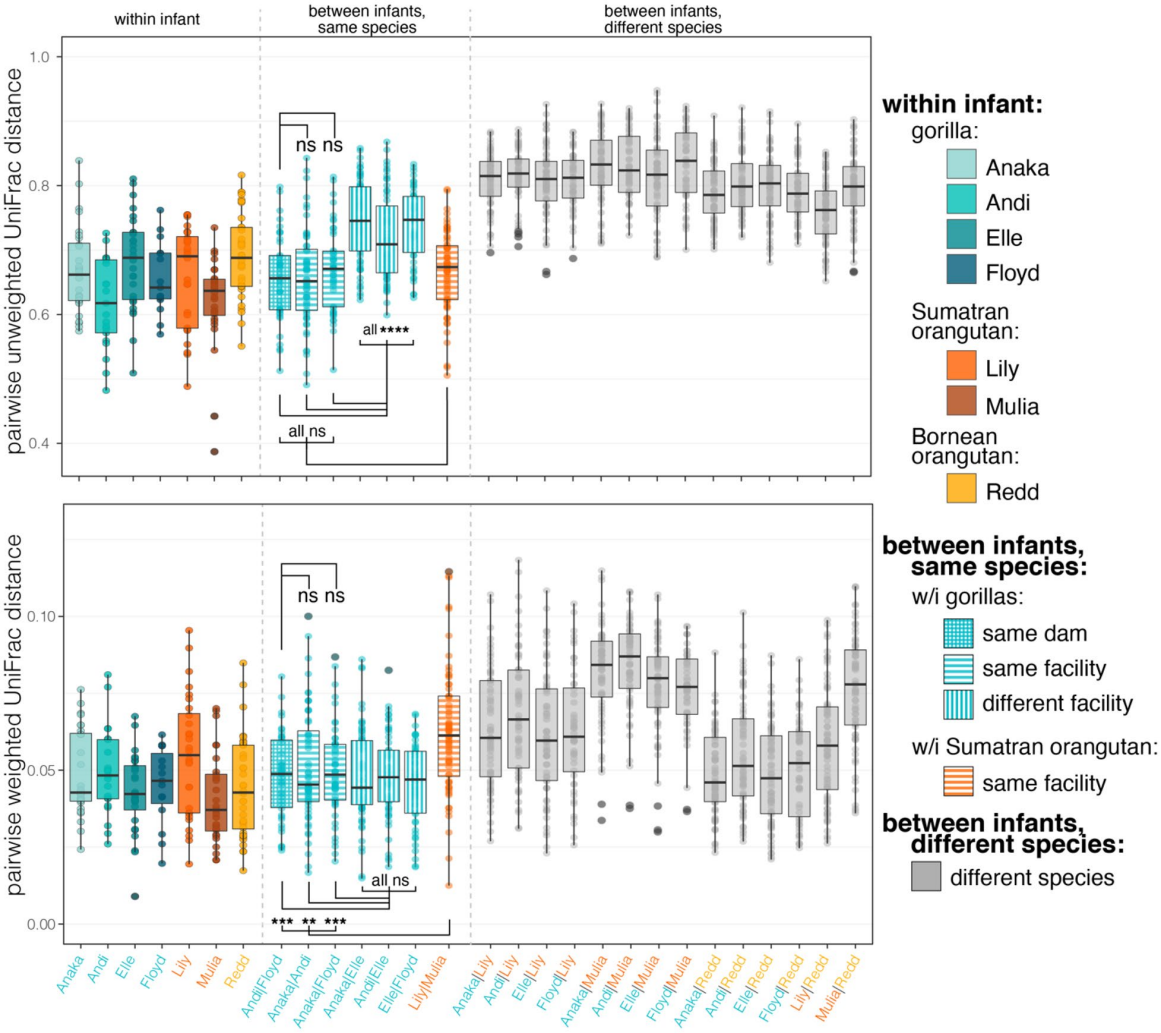
Pairwise unweighted and weighted UniFrac distances for the milk microbiomes received by gorilla, Sumatran orangutan, or Bornean orangutan infants. Each point represents a distance between two milk samples. Shows distances between samples within an infant, and between infants of the same and different species, coded by color. Hatching patterns reflect infants from the same dam or those housed at the same or different facilities. Tukey-style box and whiskers show the median (center horizontal line) and the interquartile range (upper and lower bounds of the box), with outliers that are 1.5 times less than the 25th quartile or 1.5 times more than the 75th quartile. Statistical results calculated with Pairwise Wilcoxon Rank Sum Tests with Benjamini–Hochberg adjustment (p > 0.5 = ns; p < 0.01**; p < 0.001***; p < 0.0001****). Statistical results of insterest are shown in the figure; see Table S1 for full statistical results.

Among the gorillas, the effect of housing facility was most evident in the composition of less abundant taxa. In presence-absence composition, gorilla milk microbiomes were more similar when comparing individuals at the same facility (within Zoo Atlanta) versus when comparing individuals at different facilities (Zoo Atlanta vs. Cincinnati Zoo) (Fig. 8; Pairwise Wilcoxon rank-sum tests with Benjamini–Hochberg adjustment: UUF distances between gorillas at the same facility (${\overline{\text{x}}}$ = 0.662) vs. between gorillas at different facilities (${\overline{\text{x}}}$ = 0.734), p < 0.0001). Within the gorillas housed at Zoo Atlanta, milk microbiomes were similar in presence-absence composition regardless of dam (Fig. 8; Pairwise Wilcoxon rank-sum tests with Benjamini–Hochberg adjustment: UUF distances between Zoo Atlanta gorilla lactations from the same dam (${\overline{\text{x}}}$ = 0.655) vs. Zoo Atlanta gorilla lactations from different dams (${\overline{\text{x}}}$ = 0.662), p = 0.759). Housing facility and dam had no effect on the abundance-weighted composition of gorilla milks (Fig. 8; Pairwise Wilcoxon rank-sum tests with Benjamini–Hochberg adjustment: WUF distances between gorilla lactations at the same facility (${\overline{\text{x}}}$ = 0.050) vs. between gorilla lactations at different facilities (${\overline{\text{x}}}$ = 0.047), p = 0.405).

When comparing the bacterial membership of the gorilla milks between facilities, 286 ASVs were unique to the Cincinnati Zoo gorilla whereas 883 were unique to gorillas at Zoo Atlanta; however, none of these taxa were abundant, with each accounting for less than 1% of the sequences from its respective sample.

## Discussion

Our results on great ape milk microbiomes demonstrate (i) inter-specific variation, (ii) longitudinal changes in diversity, composition, and membership that can depend on the measure of diversity/composition and on the host species, and (iii) that specific aspects of intra- and inter-specific variation can be driven by abundant or rare microbial taxa. Our findings allow for preliminary interpretations of some potential functions of the bacteria found in ape milk.

We show that longitudinal variation and environmental setting may be mediating distributional changes in the less abundant members of milk microbial communities, while host species identity seems to underlie more foundational differences in milk microbiome structure. Our primary focus was to characterize longitudinal changes in primate milk microbiomes but given that we had mothers from multiple species, we first characterized host species differences, albeit with a limited number of individuals per host species. Even with limited sample size we found support, similar to our previous research with more robust sample size per host species^22^, that host species can affect the diversity and composition of bacteria found in primate milk. We found that while certain abundant taxa were conserved across host species, variation in the less abundant microbes differentiated many of the species-specific patterns. We additionally found patterns that mirror those seen in other NHP and human milk, such as similar abundant bacterial members (i.e., dominant *Streptococcus* and *Staphylococcus* genera with minimal presence of Bacteroidetes members) and changes in bacterial composition, which suggest that certain aspects of milk microbiomes may be conserved across primates^22,37^. These results indicate that milk microbiomes are shaped by a combination of endogenous (i.e., host species) and exogenous (i.e., environment) factors, similar to results found for gut microbiomes^52,53^. Our findings reinforce the concept that milk microbiomes are not the result of incidental contamination but are a consistent and selective input of microbes into milk which likely affect neonatal microbial communities.

Despite the phylogenetic closeness of the three study species, particularly the two orangutan species, their milk microbiomes were distinct in several measures of diversity and composition. Microbial phylogenetic diversity—which does not account for microbial abundance—differed significantly between species. Similarly, presence-absence composition, which does not account for abundance, showed more distinct clustering by species compared to the abundance-weighted measure. In combination, these results demonstrate that less abundant bacteria were important drivers of species differences in milk microbiomes; when abundant microbes were given more weight, the milk microbiomes were more similar in diversity and composition, a pattern that was likely driven by a few shared, high-abundance taxa. Unlike the gut microbiomes of NHPs, which can have high representation of members from the *Bacteroidetes* phylum, members of the genera *Streptococcus* and *Staphylococcus* dominated the milk microbiomes of all three species, accounting for almost half of all sequence reads. *Streptococcus* and *Staphylococcus* are also found in high abundances in human milk microbiomes^32^ and include well-studied pathogenic members^54,55^, such as *Staphylococcus aureas*. However, whether these members of the two genera function as detrimental pathogens in the milk microbiome or infant gut community remains unknown. Moreover, the members of these genera are phylogenetically similar and notoriously difficult to distinguish at the strain level^56–58^. The high abundance and phylogenetic similarity of *Streptococcus* and *Staphylococcus* taxa were likely drivers of the patterns seen in abundance-weighted measures of diversity and phylogenetic composition.

One of the four abundant ASVs that was shared across all three host species, identified as *Streptococcus parasanguinis*, (i) was present in every sample in this study, (ii) has been found to be a dominant microbe in human milk microbiomes^57^, and (iii) was identified as a “core operational taxonomic unit (OTU)” (99% sequence identity match between ASV sequence and OTU representative sequence^22^) in our previous study of nine primate species which included catarrhine and platyrrhine monkeys. In human milk microbiomes, the abundance of *S. parasanguinis* decreases over time, mirroring the patterns seen in this study^27^. These results suggest that *S. parasanguinis* may be a ‘keystone’ member of anthropoid primate milk microbiomes. *Streptococcus parasanguinis* is also widely known as a member of the human oral microbiomes and has been suggested as a pioneer member of infant oral cavities^59^. These patterns suggest that there may be continuous, bidirectional transmission of *S. parasanguinis* between the mammary gland and infant oral microbiomes.

Great ape milk microbiomes changed over time in Shannon diversity, but showed species-specific patterns. Contrary to our prediction that all alpha diversity measures would not vary significantly over time, we found that Shannon diversity increased over time in gorillas and the Bornean orangutan. Interestingly, this pattern is consistent with findings in vervet monkeys, where Shannon diversity increased in later lactation^37^. However, we also found that the other measures of alpha diversity stayed relative stable over time, supporting our previous findings in gorillas and Sumatran orangutans, where bacterial richness did not vary significantly over time^22^. In Sumatran orangutans, however, Shannon diversity stayed relatively stable. Measures of bacterial alpha diversity within an individual’s milk microbiome are known to vary over time^22^. Variation in longitudinal sampling regimes may underpin the different patterns we observed compared to the other, few studies of NHP milk microbiomes and even across the limited number of individuals within our own study. Many previous studies have demonstrated microbial differences between the extremes of lactation stages (e.g., early or late lactation vs. mid-lactation^25,32,34,36^) but few have examined the more nuanced variation in milk microbes across a large range of the host’s entire lactation period. In addition, our approach of fitting trajectories of microbial variation to longitudinal time points, and not examining the microbiomes by binning into lactation stage categories, is less sensitive to variation in sampling regime. We emphasize that balanced longitudinal sampling would provide much-needed resolution on the interspecies variation in milk microbiomes over time.

Longitudinal variation in microbial structure was detected in presence-absence composition, which gives equal consideration to rare and abundant taxa, but it was not detected in abundance-weighted composition, which gives more weight to variation in abundant taxa. This pattern suggests that changes in the rarer taxa, more so than the abundant taxa, were driving the longitudinal variation in this study. We previously found similar patterns of change in presence-absence composition, but also in abundance-weighted composition in gorillas, but not in Sumatran orangutans^22^. Our previous study was limited in that we were not able to sample both early and mature milks for more than a 300-day span in lactation for most individuals, reemphasizing that sampling across multiple lactation stages can be critical for detecting longitudinal changes in milk microbiome composition. In our present study, the majority of abundant taxa did not vary in abundance over time, suggesting a mechanism that maintains their presence and abundance. Of the few that did vary significantly over time, the majority decreased in abundance, possibly leaving open niches for colonization by more diverse, less abundant microbes (i.e., as reflected in the longitudinal increase in diversity). These results emphasize the importance of considering both rare and abundant taxa as they may show different biologically relevant patterns.

Our data also allowed for inferences into potential functions of milk microbes. In all primates, milk oligosaccharides are an important resource for many of the dominant microbes found in milk microbiomes. In humans, diversity and concentration of milk oligosaccharides decreases across lactation^60^, but remains exceptionally high compared to another mammals^61–63^. Members of the *Streptococcus* and *Staphylococcus* genera are known to ferment oligosaccharides, including in dairy products^64,65^. *Streptococcus thermophilus*, for example, produces enzymes that break down milk oligosaccharides and galactooligosaccharides; however, its hydrolytic activity is less pronounced than that of Bifidobactria^64,66,67^. Considered a promising probiotic in humans, *Streptococcus thermophilus* also produces antimicrobials that prevent gut inflammation and pathogen-induced diarrhea^68^. In this study, *Streptococcus thermophilus* is shared across the three species and decreases significantly over time, reflecting a potential early role in milk sugar digestion with possible consequences for inflammation and pathogen protection in young ape infants.

Longitudinal change in the abundances of specific bacteria may also reflect selective pressures driven by mother and infant immune systems. The naïve immune systems of young infants require microbial metabolites to regulate inflammation and develop appropriate immune responses^10,69^. A recent study in human infants demonstrated that a lack of *Bifidobacterium* and, specifically, the microbial genes that code for oligosaccharides utilization, resulted in systemic inflammation and general immune dysregulation in the developing infant^10^. Administration of *Bifidobacterium* restored immune function and reduced systemic inflammation^10^. Although *Bifidobacterium* were largely absent from the ape milk microbiomes in this study, it is likely that other bacteria may serve similar functions to break down oligosaccharides, as mentioned above. Immune processes may initially select for these sugar-degrading microbes to kickstart infant immune development^70^. Similarly, milk microbes may act to train the infant’s immune system to tolerate specific, incoming microbes from maternal microbiomes, facilitating the recognition and tolerance of commensal or symbiotic microbes as members of ‘self’ as opposed to foreign antigens^10,71^. Further investigation of these selective pressures and microbial functions requires increased metagenomic and immune study in NHP mothers and infants.

Although our study was not designed to specifically address the influence of housing facility, we found evidence for a limited effect of facility on the patterns we detected within gorilla milk microbiomes. This supports previous findings that environmental conditions, including diet and exposure to environmental microbes, can shape milk microbiomes^72^ and NHP gut and skin communities^73–75^. In the case of the gorilla milk microbiomes in this study, housing facility influenced the less abundant taxa enough to differentiate members of the same host species housed at different locations. This was, again, evident when comparing results of presence-absence and abundance-weighted composition: the influence of housing facility was only discernible in presence-absence, the metric that is more sensitive to rare taxa. Although we cannot pinpoint the precise factors underlying this environmental mediatization of less abundant taxa, these results motivate further research into the influence of housing facility, geographic location, and environmental variation^41^ on intraspecific variation in milk microbiomes.

In conclusion, we provide an evaluation of the bacteria in the milk of three species of great apes over time. We found that ape milk microbiomes are shaped by host species identity but have the dynamic ability to shift over time and are affected by proximate cues likely originating from mother, offspring, and the environment. Despite the limitations of our small number of individuals for each host species, we found microbial differences between host species, highlighting the strong role of evolutionary history in determining milk microbiome structure and mirroring patterns seen in other microbiomes^53,76,77^. We found nuanced longitudinal changes in milk microbiome diversity, composition, and membership. These changes may reflect shifting resource availability and potential selection for immune-related microbial functions. Despite the strong influences of host species, signals of different housing facilities were discernible in the less abundant bacteria. Our data reflect both expected and novel patterns in NHP milk microbiomes that reinforce and expand existing frameworks on the factors that shape reproductively relevant microbiomes. Moreover, these data provide important initial insights for further investigations of the functional roles of milk microbes in NHP infant health and development, a key component of NHP husbandry and conservation.

## Supplementary Information

Supplementary Information.

## Data Availability

Bioinformatic pipeline, statistical scripts, and appropriate metadata are available in Open Science Framework (OSF Project: osf.io/vyxr7). Raw 16S sequencing data are available from NCBI’s Sequence Read Archive (BioProject ID PRJNA838933).
